# Adaptation of the Childbirth Experience Questionnaire (CEQ) in China: A multisite cross-sectional study

**DOI:** 10.1371/journal.pone.0215373

**Published:** 2019-04-24

**Authors:** Xiu Zhu, Yan Wang, Hong Zhou, Liqian Qiu, Ruyan Pang

**Affiliations:** 1 Dept. of Maternal and Child Health, School of Public Health, Peking University, Beijing, China; 2 School of Nursing, Peking University, Beijing, China; 3 Dept. of Women Health, Women Hospital, School of Medicine, Zhejiang University, Hangzhou, China; 4 Chinese Maternal and Child Health Association, Beijing, China; Academic College of Tel Aviv-Jaffa, ISRAEL

## Abstract

**Background:**

The childbirth experience of women represents a significant aspect of quality care. Due to the lack of a reliable Chinese language tool for assessing childbirth experiences, examples must be adapted from other countries. The aim of this study was to translate an English version of the Childbirth Experience Questionnaire (CEQ) into Chinese and adapt this tool to the Chinese context.

**Methods:**

A questionnaire validation study was conducted. A forward-backward translation procedure involving the developer of the CEQ was conducted. The data were collected in postnatal wards at 50 birth facilities in 4 regions of Zhejiang Province, China. Women who gave birth vaginally at the investigated facilities during the study period completed an online questionnaire that included the Chinese version of the CEQ (CEQ-C), demographic information and clinical information. Psychometric analyses were performed to assess the internal and content consistency. After subdividing the sample into subsamples, an exploratory factor analysis (EFA) and confirmatory factor analysis (CFA) were applied to examine the structural validity. Known-group comparisons were performed to assess the discriminant validity.

**Results:**

Overall, 1747 women participated in this study. The content validity index (CVI) of the CEQ was 0.92. Based on the comments of the experts combined with the statistical results, we removed 3 items related to pain, sense of control and sense of security and changed 3 items to different dimensions. The CFA supported the four dimensions of the CEQ-C (standard root mean square residual (SRMR) = 0.037, root mean square error of approximation (RMSEA) = 0.036, comparative fit index (CFI) = 0.966, and Tucker-Lewis index (TLI) = 0.959). Cronbach’s alpha of the CEQ-C was 0.88, and McDonald’s omega value was 0.91. The duration of labor, delivery mode, parity, oxytocin augmentation, pain management, companionship, prenatal education and pain experienced exerted significant effects on the women’s childbirth experiences.

**Conclusions:**

Although some items performed differently in our analysis comparing the English and Chinese versions of the CEQ, the CEQ-C is reliable and valid. Additionally, the CEQ-C is an easy-to-use and promising tool for measuring childbirth experiences among Chinese women in facility settings that can be used to improve the quality of intrapartum care. Efforts are needed to provide women with respectful, evidence-based intrapartum care to facilitate positive childbirth experiences.

## Introduction

As progress in reducing maternal mortality is achieved, a new and broader focus encompassing the quality of maternity care is needed.[1] This need is particularly relevant in areas with low maternal mortality and high coverage in terms of antenatal care and facility childbirth, where the content of care (insufficient or excessive intervention) and women’s satisfaction should take precedence.[2] Women’s experiences with childbirth represent a vital outcome indicator of intrapartum care and have both immediate and long-term effects on future reproduction.[3,4] Person-centered care for positive childbirth experiences is currently a global trend, and the WHO has issued guidelines recognizing a “positive childbirth experience” as a significant end point for all women undergoing labor.[5] An understanding of this issue may help clinicians identify maternal needs and aspects of care that potentially require improvement.

China has achieved substantial progress in reducing maternal and infant mortality, which is largely attributed to the increase in facility childbirths.[6,7] However, many facilities fail to provide high-quality or respectful care,[8] and researchers appear to have neglected this field of study. To date, only one study has qualitatively assessed the expectations and experiences of a small group of women in rural China, revealing the inefficient provision of quality care during childbirth.[8] Furthermore, no reliable tool for assessing the quality of childbirth care is available in Chinese.

Various instruments measuring childbirth experiences are available in multiple languages. Among these instruments, the Childbirth Experience Questionnaire (CEQ),[9] which was created in Swedish by Dr. Dencker et al. in 2010, is one of the most valid and reliable tools according to a thorough comparison of multiple instruments in this field[4] and has been transculturally validated in English and Spanish.[10,11] The CEQ assesses women’s childbirth experiences in four dimensions, i.e., *own capacity*, *professional support*, *perceived safety* and *participation*, which conforms to the philosophy of normal childbirth.[12] Currently, the CEQ is widely used to identify women with negative childbirth experiences and evaluate efforts to improve the quality of childbirth care.[9–11,13] We chose the CEQ because it is the most recently published multidimensional instrument that comprehensively evaluates women’s perceptions and feelings.

The CEQ is recommended for use in community settings among mothers within one month of childbirth to recall their experiences at a birth facility.[9] However, researchers and clinicians have questioned whether the CEQ should be applied in facilities to provide a more timely assessment of the quality of care. Hence, this study was conducted to adapt the CEQ to the Chinese context, assess its psychometric properties (validity and reliability) and further adjust this instrument for Chinese women to assess their childbirth experiences.

## Methods

This study consisted of two phases. In Phase I, a Chinese version of the CEQ (CEQ-C) was produced, and 94 postpartum women completed two pretests (34 women in the first round and 60 women in the second round) to test its content validity. In Phase II, a field study involving 1747 women was conducted to test the construct validity and reliability.

### Phase I: Translation of the CEQ and pretest

A forward-backward translation procedure[14] involving the developer of the CEQ was conducted. First, we obtained permission to adapt and use the CEQ from Dr. Dencker, who was the creator of the original Swedish version of the CEQ, and Dr. Walker, who was the author of the English version of the CEQ. Second, the English CEQ was forward translated into Chinese by two female bilingual translators who are native Chinese-speakers (one translator is a Master’s student studying at an American university, and the other translator is a senior program officer whose work language is English). Then, both translators compared their translations, reached a consensus regarding the semantic, idiomatic, and conceptual equivalence, and produced a single draft of the Chinese version of the questionnaire. Subsequently, cognitive interviews were conducted with 34 postpartum women to test the comprehensibility and legibility of the questionnaire, and the questionnaire was revised accordingly. Then, two other female bilingual translators who are native Chinese speakers and were completely blinded to the CEQ translated the Chinese version back into English, and this translation was compared with the English version of the questionnaire generated by the creator of the CEQ; no discrepancies were noted. Hence, the final Chinese version of the CEQ (CEQ-C) was prepared. Then, 60 postpartum women from two postnatal wards in Zhejiang Province were informed about the study and invited to complete the CEQ-C questionnaire online before they were asked certain questions in a face-to-face interview concerning the ease of completion, time required and acceptability of the questionnaire from their perspectives.

### Phase II: Field study

#### Study sample

The study was conducted at 50 health institutes in four regions of Zhejiang Province between July and October 2017. Zhejiang is a relatively wealthy coastal province in Eastern China. The provincial cesarean section rate was 44.0% in 2014.[15] The four regions included one of the two provincial level cities and 3 prefecture-level cities that were selected from the 3 categories of cities stratified by the number of annual childbirths. The included health institutes agreed to participate in the study. The women who delivered babies at these institutes were recruited from the postnatal wards before discharge (2–3 days after childbirth). The inclusion criteria were: (a) age >18 years; (b) vaginal delivery; (c) ability to read Chinese; and (d) willingness to participate in the study. All participants provided oral consent to complete the study. During the study period, 1,832 postpartum women were approached, and 1,747 participants completed the entire online questionnaire and were included in the study. The response rate was 95.3%.

#### CEQ-C

The CEQ-C contains 22 items that measure the following four main dimensions of the childbirth experience: *own capacity* (8 items), *professional support* (5 items), *perceived safety* (6 items) and *participation* (3 items). The responses to 19 items were scored using a 4-point Likert scale ranging from 1 (totally agree) to 4 (totally disagree). Three items referring to pain, sense of control and sense of security were assessed with a visual analog scale (VAS) on which zero indicates no pain/control/security and 100 indicates the worst imaginable pain/complete control/feeling totally secure. The scores of the negatively worded items were reversed, and the VAS values were transformed to categorical values for scoring (0–40 = 1, 41–60 = 2, 61–80 = 3 and 81–100 = 4). The item ratings were aggregated to scale scores by summing the coded values of the items on each scale, followed by dividing by the number of items on the scale (i.e., to obtain the mean). The score ranged from 1 to 4 points, and higher ratings reflected more positive experiences.

#### Data collection

The CEQ-C was administered online along with demographic and clinical questions through the Wenjuanxing web platform (http://www.wjx.cn). The demographic information included age and educational background. The clinical information included parity, onset and duration of labor, type of delivery, augmentations, pain management, complications, continuous electronic fetal heart rate monitoring (EFM), companionship, and prenatal education.

At the beginning of the field study, a nurse at each health institute who did not work in the labor and delivery room received face-to-face instructions from the designated researcher on the methods to recruit the women, proceed with obtaining informed consent and instruct the women to complete the anonymous questionnaire. Fifty nurses from 50 health institutes participated in the data collection process. Once the women were ready for discharge (two or three days after giving birth), the nurses provided information about the study and gave the women a card with a two-dimensional code picture. Then, the women were able to freely complete the questionnaire on their smart phones before discharge. The staff at the birth institutes were blinded to the information that the women provided. Because the two-dimensional code was only able to be scanned at the facilities, the women were only able to complete the questionnaire before they were discharged. All procedures were conducted in the same manner at all 50 sites.

#### Statistical and psychometric analyses

The psychometric properties of the CEQ were assessed according to the COSMIN checklist standards.[16]

The validity of the CEQ-C was assessed according to five aspects. (a) The content validity, which describes whether an instrument adequately covers the dimensions to be evaluated, was assessed by determining the face validity and the content validity index (CVI) in this study.[16,17] We invited five experts from different fields to evaluate the CVI of the CEQ-C. These experts included a senior midwife, an obstetrician, a university professor in obstetric nursing and two senior researchers in the maternal health field (one of whom had a psychology background). The experts were asked to rate each item on a 4-point scale ranging from 1 to 4 (1 = not relevant, 2 = somewhat relevant, 3 = quite relevant, and 4 = highly relevant) according to the applicability of the expression and content to the local culture and the research objective. The CVI of each item (I-CVI) was calculated as the ratio of the number of “quite relevant” and “highly relevant” expert opinion responses to the number of experts.[18] The overall CVI of the questionnaire was calculated as the average of the I-CVIs of all items. Items with an I-CVI somewhat lower than 0.78 were considered candidates for revision, and items with very low values were candidates for deletion.[17] A CVI rating greater than 0.8 represented satisfactory content validity. (b) Floor and ceiling effects, which involve range restrictions at the lower and upper ends of a measure, respectively, have been recommended to be lower than 15%.[19,20] Items with high floor/ceiling effects were considered for removal.[21,22] (c) An item-total correlation test was performed to determine whether any item in the set of tests was inconsistent with the average behavior of the other items and thus could be discarded. The standard recommendation is to eliminate items whose item-total correlation is less than 0.30.[21] (d) The structural validity of the questionnaire was assessed using an exploratory factor analysis (EFA) and confirmatory factor analysis (CFA).[23–25] (e) The discriminant validity was determined based on differences in the CEQ-C scores among subgroups known to differ in key variables,[9] including duration of labor, delivery mode, parity, oxytocin augmentation, pain management, companionship, prenatal education and pain experienced. As the scores were not normally distributed, the Mann-Whitney *U* test and Kruskal-Walls *H* test were used to compare the scores among the groups. A *P-*value less than 0.05 was considered significant.

The reliability of the CEQ-C was explored by examining the following three indicators of internal consistency: (a) Cronbach’s alpha coefficient, where an internal consistency value greater than 0.7 is considered satisfactory;[18] (b) McDonald’s omega value,[26,27] where a value above 0.70 indicates reliability; and (c) decreased alpha values as individual items were removed.[28]

Regarding the adaptation of the questionnaire, a CFA of all items was first conducted. As the CFA failed to fit the factor structure in the original instrument, an EFA was used to improve the model. The samples were divided into two groups, and an EFA with maximum likelihood estimation and promax rotation for the first half of the samples was applied to identify the number and structure of the underlying constructs. The Kaiser rule (eigenvalue>1.0) was applied to determine the number of dimensions to extract. The acceptable factor loading was set to greater than 0.4. The research team reviewed and discussed the EFA results and jointly selected a factor structure for further analysis, which was applied to the other half of the samples for a CFA to assess the fit of the factor structure. A good model fit was confirmed by a standardized root mean square residual (SRMR) ≤0.08, root mean square error of approximation (RMSEA) ≤0.06 and comparative fit index (CFI) and Tucker-Lewis index (TLI) ≥0.95.[29]

The statistical analyses were conducted with SPSS 20 (SPSS Inc., Chicago, IL, USA), Mplus 7.4 and R X64 3.5.1 (*psych* package).

### Ethics

The study received ethical approval from the Institutional Review Board of Peking University Health Science Center on 19 July, 2017: Reference: IRB00001052-17067. Oral informed consent was obtained from all participants based on a standardized consent script, which was approved by the Institutional Review Board of Peking University Health Science Center. The reason for obtaining oral consent instead of written informed consent was that the only record that the participant was contacted regarding the study was the informed consent document. Oral consent was manually documented by a member of the research team.

## Results

### Respondent characteristics

One thousand seven hundred forty-seven women (mean age: 27 years; range 18–47 years) were included in the study. Over half of these women were primiparous (53.3%), and most women underwent spontaneous vaginal delivery (93.0%), exhibited a labor duration of less than 12 h (79.3%) and had company during labor (89.8%). More than 90% of the participants experienced no complications during pregnancy and labor. Approximately 27% of the participants attended prenatal education sessions, 41% of the participants received augmentation during labor, 63% of the participants did not use any pain relief method, and 93.1% of the participants used continuous EFM (Table 1).

**Table 1:** Demographic and clinical characteristics of the study population (n = 1,747).

**Variables**	**N (%)/M (SD)**	**Variables**	**N (%)**
**Education**		**Oxytocin augmentation**	
** High school or below**	828 (47.4)	**No**	879 (53.0)
** College or above**	919 (52.6)	**Yes**	728 (41.7)
**Maternal age, years, mean (SD)**	27 (4.00)	**Unknown**	140 (8.0)
**Gestational age, weeks, mean (SD)**	39 (1.65)	**Prenatal education**	
**Previous deliveries**		**No**	1,273 (72.9)
** No**	932 (53.3)	**Yes**	474 (27.1)
** Yes**	815 (46.7)	**Pain management**	
**Onset of labor**		**No pain relief method**	1,108 (63.4)
** Spontaneous**	1,138 (65.1)	**Pharmacy pain relief**	399 (22.8)
** Induction**	469 (26.8)	**Nonpharmacy pain relief**	188 (10.8)
** Unknown**	140 (8.0)	**Unknown**	52 (3.0)
**Labor duration less than 12 h**	1,229 (79.3)	**Companionship**	
**Type of delivery**		**Yes**	1,568 (89.8)
** Spontaneous vaginal**	1,625 (93.0)	**No**	179 (10.2)
** Instrumental**	122 (7.0)		

### Validity

#### Content validity

The pretests revealed a good content validity of the CEQ-C. Regarding face validity, the translated version was acceptable and understandable to women, easy to complete, and required an average of 15 minutes to complete. Sentences were added to the instructions to adapt the instrument to the Chinese healthcare context (which defines labor and delivery as the period “from entering the labor room to being transferred to the postnatal ward after giving birth to a baby”). We added “doctor and nurse” after “midwife” to some items; for example, the item “My midwife understood my needs” in the original version was changed to “My midwife (doctor or nurse) understood my needs” in the CEQ-C. We defined the birthing position to include “kneeing, squatting or lying in bed” in the CEQ-C, as some women did not know how to answer the question “I felt that I could have a say in deciding my birthing position” in the pretest. In general, three items (items 20, 21 and 22) had I-CVIs of 0.6, three items (items 5, 7 and 18) had I-CVIs of 0.8, and the CVI of the CEQ-C was 0.92. Although the CVI was acceptable, 3 items (items 20, 21 and 22) were disputed: two experts responded “not relevant” to these three items and suggested the deletion of these items, and three experts responded “quite relevant” to these three items with some comments.

#### Structural validity

After applying the original 4-factor model to the CFA (SRMR = 14.12, RMSEA = 0.072, CFI = 0.372, and TLI = 0.286), the results indicated that an EFA was needed to modify the model of the CEQ-C (Table 2). The items and data were carefully analyzed, and the decision was made to eliminate items 20 (pain), 21 (sense of control) and 22 (sense of security) to improve the construct validity. This decision was based on the following evidence: 1) these three items presented the highest ceiling/floor effects (77.9%, 47.5% and 71.5%, respectively) (Table 3); 2) these items were at the lowest end of this range (0.186, 0.357 and 0.308, respectively) (Table 3); 3) these three items had the lowest I-CVIs (0.6); and 4) the experts suggested the removal of these items, as the information represented by items 21 and 22 was captured in items 19 (addressed the situation well) and 18 (felt secure).

**Table 2:** Goodness-of-fit of the indicators in the original CEQ factor model and CEQ-C factor model.

Index	CEQ*	CEQ-C**	Standard cut-off values
**GFI**	0.895	0.953	>0.9
**AGFI**	0.869	0.937	>0.9
**RMSEA**	0.072	0.036	<0.05
***P* value**	<0.001	<0.001	<0.05
**CFI**	0.372	0.966	>0.9
***X*^2^/df**	9.93	5.53	0–1
**NNFI (TLI)**	0.286	0.959	>0.9
**SRMR**	14.12	0.037	<0.05

*The original model defined according to the conceptual structure of the original study. The following items were included in each dimension: “own capacity” (items 1–2, 4–6, and 19–21), “professional support” (items 13–17), “perceived safety” (items 3, 7–9, 18, and 22), and “participation” (items 10–12).

**The model was adjusted according to the EFA. Items 19–22 were removed, and the locations of items 5, 7 and 18 were changed.

**Table 3:** Information regarding the performance of the items (n = 1,747).

Items	Mean±SD	Item total correlation values	Floor and ceiling effect [n (%)]
**1. Labor progress went as I had expected**	3.00±0.64	0.635**	355 (20.3)
**2. I felt strong**	2.98±0.59	0.632**	284 (16.2)
**3. I felt scared ^R^**	2.27±0.67	0.381**	217 (12.4)
**4. I felt capable**	2.93±0.55	0.619**	210 (12.0)
**5. I felt tired ^R^**	2.01±0.59	0.374**	292 (16.7)
**6. I felt happy**	2.74±0.72	0.617**	289 (16.5)
**7. I have many positive memories**	2.80±0.71	0.650**	295 (16.8)
**8. I have many negative memories ^R^**	2.79±0.65	0.498**	218 (12.4)
**9. I felt depressed ^R^**	2.65±0.68	0.489**	205 (11.7)
**10. I could choose whether to be up and moving or lying down**	2.88±0.60	0.442**	228 (13.1)
**11. I could choose the delivery position**	2.34±0.72	0.406**	252 (14.4)
**12. I could choose the pain relief method**	2.80±0.62	0.401**	197 (11.3)
**13. My midwife devoted enough time to me**	3.10±0.59	0.673**	397 (22.7)
**14. My midwife devoted enough time to my partner**	3.01±0.61	0.673**	334 (19.1)
**15. I was kept informed**	3.15±0.54	0.659**	400 (22.9)
**16. My midwife understood my needs**	3.10±0.54	0.720**	351 (20.1)
**17. I felt very well taken care of by the midwife**	3.18±0.55	0.681**	450 (25.8)
**18. I felt secure**	3.20±0.50	0.662**	432 (24.7)
**19. The situation was well handled**	2.82±0.65	0.697**	238 (13.6)
**20. Labor pain, VAS^R^**	1.39±0.75	0.186**	1,354 (77.5)
**21. Sense of control, VAS**	2.81±1.05	0.357**	837 (47.9)
**22. Sense of security, VAS**	3.55±0.76	0.308**	1,257 (71.9)

^R^ Ratings of negatively worded statements are reversed.

**P<0.001

Then, the samples were randomly divided into two groups, and an EFA was applied to the first half of the samples to raise the adjusted model of the CEQ-C. Bartlett’s test was significant (*X*^2^ = 1469, df = 171, P<0.001), and the Kaiser-Meyer-Olkin (KMO) value was 0.907. The four dimensions (Table 4) were extracted according to the standard that the loading of each factor should be greater than 1, and the dimensions accounted for 49.97% of the total variance. According to the results, the locations of items 5 (“I felt tired”) and 7 (“I have many positive memories”) were exchanged; item 18 (“My impression of the medical competence made me feel secure”) was moved from “*perceived safety*” in the original version to “*professional support*”. After the EFA, a CFA of the other half of the samples was conducted based on the adjusted model, and the results confirmed the four-factor structure with a good model fit (SRMR = 0.037, RMSEA = 0.036, CFI = 0.966, and TLI = 0.959) (Table 2).

**Table 4:** Exploratory factor analysis (EFA) results.

	**Dimensions**			
**Items**	1	2	3	4
**16. My midwife understood my needs**	0.848			
**17. I felt very well taken care of by the midwife**	0.835			
**15. I was kept informed**	0.829			
**13. My midwife devoted enough time to me**	0.790			
**14. My midwife devoted enough time to my partner**	0.778			
**18. I felt secure**	0.771			
**8. I have many negative memories ^R^**		0.683		
**9. I felt depressed ^R^**		0.672		
**3. I felt scared ^R^**		0.586		
**5. I felt tired ^R^**		0.525		
**10. I could choose whether to be up and moving or lying down**			0.663	
**12. I could choose the pain relief method**			0.547	
**11. I could choose the delivery position**			0.473	
**2. I felt strong**				0.771
**4. I felt capable**				0.732
**1. Labor progress occurred as I had expected**				0.671
**7. I have many positive memories**				0.628
**6. I felt happy**				0.624
**19. The situation was well handled**				0.603
**Eigenvalue**	6.638	2.157	1.652	1.114
**Variance explained**	32.653	8.718	5.781	2.819
**Cumulative variance explained**	32.653	41.371	47.152	49.971

#### Discriminant validity

The discriminant validity of the CEQ-C was tested using a known-group validity assessment (Table 5). The duration of labor, delivery mode, and parity produced significant differences in the dimensions of *“own capacity”* and *“perceived safety*” and the overall CEQ-C score. The women who did not receive oxytocin augmentation during labor reported significantly higher scores for the dimensions of *“professional support”* and *“own capacity”*. Pain management also exerted a significant effect on the dimensions *“professional support”*, *“own capacity”* and *“participation”* and the overall CEQ-C score. The women who were not accompanied by family or others during labor reported lower scores for the dimensions *“professional support”*, *“own capacity”*, and *“participation”* and the overall CEQ-C score. Prenatal education and perceived pain exerted significant effects on all four dimensions and the overall scores of the CEQ-C.

**Table 5:** Differences in dimension scores and overall scores among different groups, M (SD).

Group	n	Professional support	Own capacity	Perceived safety	Participation	Overall score
**Labor duration**						
** ≤12 h**	1226	3.12(0.45)	2.91(0.45)	2.45(0.46)	2.68(0.47)	2.84(0.33)
** >12 h**	512	3.12(0.49)	2.77(0.51)	2.38(0.48)	2.65(0.49)	2.78(0.36)
P-value*		0.790	<0.001	0.004	0.074	<0.001
**Vaginal delivery model**						
** Spontaneous**	1625	3.12(0.46)	2.89(0.47)	2.44(0.47)	2.67(0.47)	2.83(0.34)
** Instrumental**	122	3.04(0.47)	2.70(0.46)	2.21(0.37)	2.64(0.52)	2.69(0.32)
P-value*		0.175	<0.001	<0.001	0.356	<0.001
**Parity**						
** Primiparous**	932	3.13(0.48)	2.83(0.51)	2.39(0.48)	2.67(0.49)	2.81(0.36)
** Multiparous**	815	3.11(0.44)	2.92(0.42)	2.46(0.48)	2.67(0.49)	2.84(0.32)
P-value*		0.054	<0.001	<0.001	0.909	0.039
**Augmentation**						
** No**	879	3.09(0.42)	2.91(0.46)	2.46(0.46)	2.66(0.47)	2.83(0.33)
** Yes**	728	3.15(0.49)	2.84(0.47)	2.40(0.47)	2.69(0.47)	2.82(0.35)
** Unknown**	140	3.14(0.51)	2.84(0.49)	2.40(0.49)	2.62(0.51)	2.81(0.35)
P-value**		0.010	0.009	0.057	0.269	0.855
**Pain management**						
** No pain relief**	1108	3.10(0.46)	2.89(0.45)	2.44(0.45)	2.64(0.47)	2.82(0.34)
** Pharmacological**	399	3.13(0.47)	2.82(0.49)	2.40(0.50)	2.71(0.48)	2.81(0.35)
** Nonpharmacological**	188	3.24(0.50)	2.89(0.53)	2.42(0.47)	2.78(0.49)	2.88(0.37)
** Unknown**	52	3.11(0.34)	2.97(0.37)	2.39(0.46)	2.60(0.43)	2.83(0.24)
P-value**		0.001	0.005	0.466	<0.001	0.033
**Companionship during labor**					
** Yes**	1568	3.13(0.46)	2.89(0.47)	2.43(0.46)	2.68(0.48)	2.84(0.34)
** No**	179	3.01(0.44)	2.75(0.49)	2.40(0.52)	2.56(0.46)	2.73(0.35)
P-value*		<0.001	<0.001	0.284	0.003	<0.001
**Prenatal education**						
** No**	1273	3.10(0.46)	2.85(0.46)	2.39(0.45)	2.63(0.47)	2.80(0.33)
** Yes**	474	3.18(0.46)	2.93(0.49)	2.52(0.50)	2.77(0.48)	2.90(0.36)
P-value*		<0.001	<0.001	<0.001	<0.001	<0.001
**Pain**						
** Extreme pain**	1298	3.10(0.46)	2.82(0.47)	2.36(0.45)	2.64(0.47)	2.78(0.33)
** Strong pain**	272	3.18(0.45)	3.01(0.40)	2.60(0.44)	2.73(0.47)	2.93(0.33)
** Moderate pain**	121	3.14(0.48)	3.04(0.47)	2.61(0.49)	2.79(0.47)	2.94(0.36)
** Slight pain**	56	3.19(0.55)	3.06(0.61)	2.73(0.58)	2.77(0.47)	2.99(0.46)
P-value**		0.002	<0.001	<0.001	0.001	<0.001

* The Mann-Whitney *U* test was performed.

** The Kruskal-Walls *H* test was performed.

### Reliability

Cronbach’s alpha of the CEQ-C was 0.88. McDonald’s omega value was 0.91. After the deletion of the “each” item, the reliability of the questionnaire decreased. Based on these results, the CEQ-C displayed satisfactory internal consistency.

### CEQ-C

The adapted CEQ-C contains 19 Likert items with scores ranging from 1 (totally agree) to 4 (totally disagree) points that measure the following four main dimensions of the childbirth experience: *professional support* (6 items), *perceived safety* (4 items), *participation* (3 items), and *own capacity* (6 items). The dimensional score is the sum of the item codes in each dimension divided by the number of items. The total score is the sum of all item codes divided by the number of all items. The scores for the scale and every dimension range from 1 to 4 points, and higher ratings reflect more positive experiences.

The overall score of the CEQ-C was 2.82, and the scores of the four dimensions were: 3.12 for “*professional support*”, 2.43 for “*perceived safety*”, 2.67 for “*participation*”, and 2.87 for “*own capacity*”.

**Fig 1:**
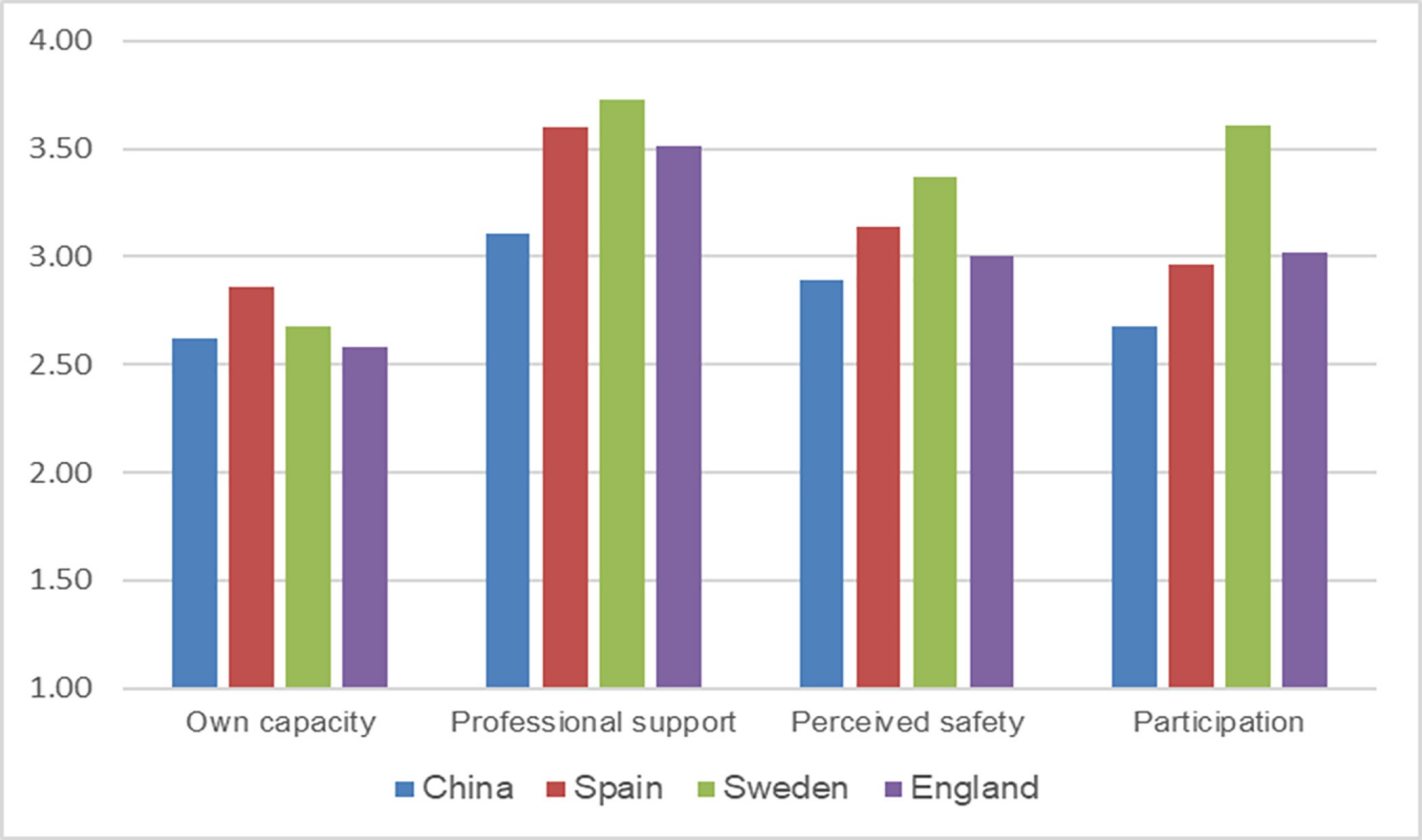
Comparison with other studies conducted in Western countries (women with a labor duration ≤12 h).

**Fig 2:**
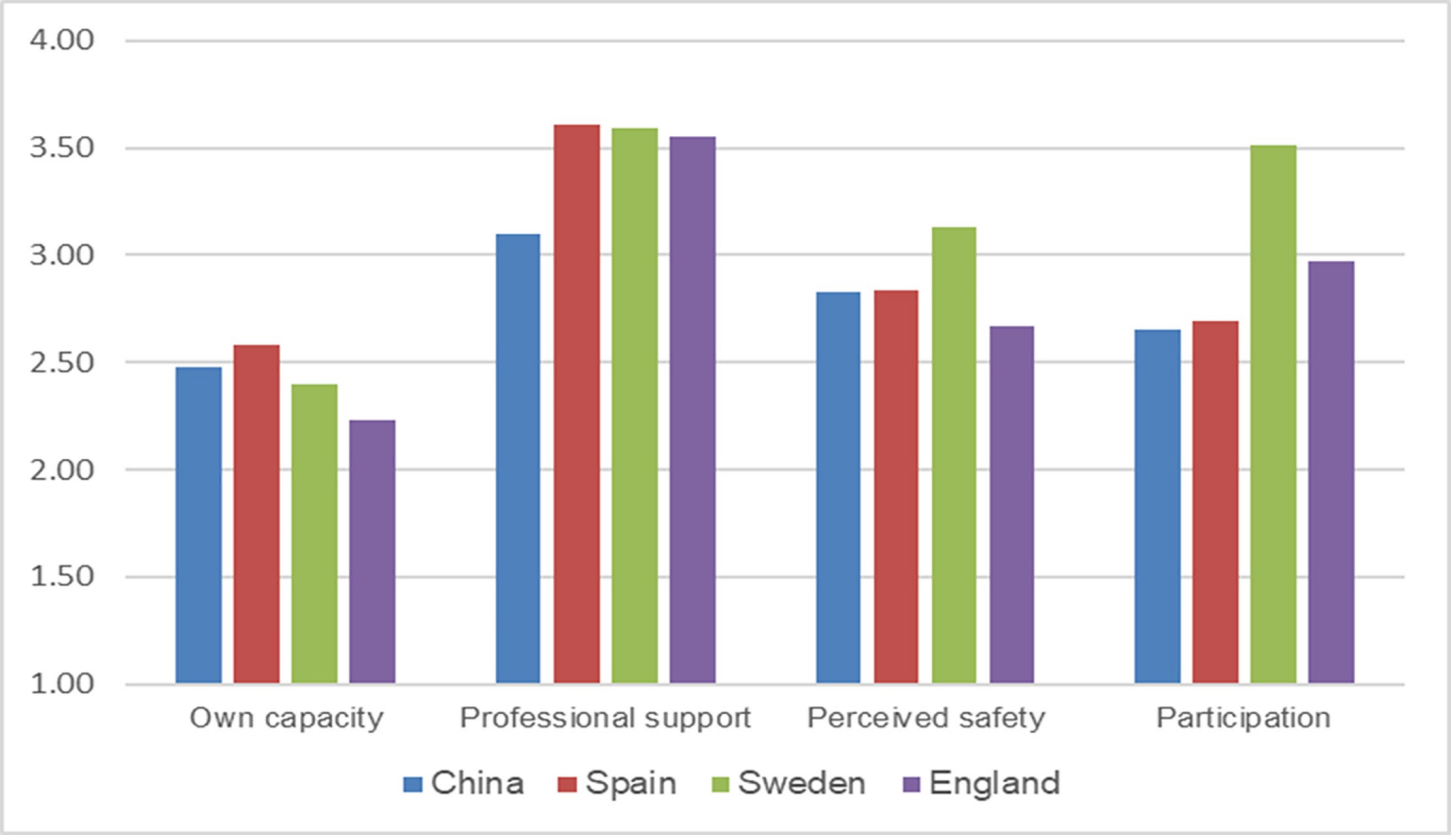
Comparison with other studies conducted in Western countries (women with a labor duration >12 h).

The results of this study were compared with findings from other available studies using CEQ measurements of maternal childbirth experiences (i.e., a comparison of our data with references from previous studies[9–11]). Since an overall score of childbirth experiences has not been reported in other studies, the same criterion (duration of labor) was selected for the comparison. The childbirth experience scores were compared between two subgroups in different studies (labor duration less than 12 h and labor duration greater than or equal to 12 h). Compared with the results obtained in other countries, the CEQ scores in our study were generally lower (Figs 1 and 2).

## Discussion

As shown in the present study, the adjusted CEQ-C is reliable and valid tool for assessing childbirth experiences among Chinese women in a facility setting. Its reliability was revealed by a Cronbach’s alpha of 0.88 and a McDonald’s omega value of 0.91, and strong correlations between the individual items and the overall score were observed. The EFA supported the four dimensions (*professional support*, *own capacity*, *perceived safety*, and *participation*), and this adjusted model was approved by the CFA.

No semantic differences existed. This result is similar to the results reported in a Spanish version adaption study.[10] In addition, little ambiguity occurred during the translation and backtranslation processes, likely because the descriptions of the 22 items in the questionnaire were relatively simple and easy to understand.

In the CEQ-C, three items (items 20, 21 and 22) were deleted, and three items (items 5, 7 and 18) were shifted to different dimensions because the factor analysis suggested that these items were not intrinsic to childbirth experiences among the Chinese population. In fact, the information conveyed by items 21 and 22 is also included in items 19 and 18, while item 20, which addresses pain, indicated a high rate of extreme pain, which is considered normal during childbirth in the Chinese culture.[8,30] Three items were transferred to maintain coherence in the content in the respective dimensions. A similar change is present in the Spanish version of the CEQ, in which item 18 was moved from *perceived safety* to *professional support*.[10] At the beginning of the content validity process, we asked the experts to mainly consider whether each item reflects the overall purpose of the evaluation, and the experts did not evaluate to which dimension each item should belong. After the results of the analysis were obtained, the experts discussed the results and thought that the dimensions of these items should be changed for consistency with the related content.

In the original CEQ, *professional support* was considered problematic in terms of the effect size, and the developers of the CEQ suggested that this issue might be due to a high ceiling effect.[9–11] However, significant differences were identified in the subgroups when additional factors other than the original four were included. The additional factors included: the pain relief method, prenatal education, companionship, and pain experienced during labor. The results further confirmed the construct validity of the CEQ.

Among the influencing factors revealed in other studies involving the CEQ, four additional factors affected childbirth experiences in China. Women with a shorter duration of labor, spontaneous vaginal delivery, multiparity and the lack of augmentation usually reported better childbirth experiences, consistent with previous studies.[9–11,13,31] In addition, the pain relief method, perceived pain, prenatal education, and companionship also potentially affected the childbirth experience.

Pain relief management and perceived pain are considered important factors contributing to the birth experiences of Chinese women. Among the study population, only 33.6% of the participants received any pain relief, including pharmacological or nonpharmacological methods. In contrast, 76% of American women are administered analgesia during childbirth, and FIGO suggests that pharmacological analgesia should be provided during childbirth to all women as desired.[32] Interestingly, nonpharmacological methods have been shown to be superior to pharmacological methods, with the latter producing no difference in the childbirth experience compared with no analgesia use; furthermore, in the dimension *own capacity*, pharmacological methods surprisingly exerted a worsening effect.

Prenatal education and companionship also affected women’s childbirth experiences. Women who attended prenatal education sessions and were accompanied by a companion during labor reported higher CEQ-C scores than other women. These findings are not surprising, as antenatal education and good companionship have been reported to improve women’s satisfaction by helping them have realistic expectations of the experience and teaching them to maintain control during labor.[33,34] However, in the present study, only 27.1% of the participants attended prenatal health education sessions. Regarding the presence of companionship, approximately 90% of the participants reported that they had someone (most commonly their husband) present during the first stage of labor. This situation was much better than that recorded in a qualitative study conducted by Raven et al.[8], in which most women who gave birth in county hospitals were not allowed to have relatives present during childbirth, and the authors concluded that women received little support from relatives in rural China. The difference in these studies concerning this issue may have been because the study by Raven was performed approximately ten years ago (between 2007 and 2009), and currently, people pay focus on companionship during labor.

Some efforts are needed to provide a positive childbirth experience to Chinese women. Compared with other studies conducted in Western countries (Sweden, the United Kingdom and Spain), Chinese women recorded lower scores on the CEQ (Figs 1 and 2). One potential explanation for this difference in scores is that the types of care offered in China differ from those offered in Western countries. These differences are evident when comparing the percentages of induced labor (41.7% in our study versus 31.7% in Spain, 21.0% in England and 13.7% in Sweden[35]). According to the proposed hypotheses, excessive interventions potentially reduce the CEQ scores. Another explanation for the lower scores in our study compared with other studies may be due to the higher percentage who did not use a pain relief method during labor in our study (63.4%). In contrast, pain relief during labor is provided to almost all women in labor in Sweden [36] through nitrous oxide (81% of women) and minimal motor block epidural analgesia (53% of primiparous women and 21% of parous women).[37]

## Strengths and weaknesses

Three main strengths of this study were identified. First, to the best of our knowledge, this study is the first to administer the CEQ in a facility setting rather than a community setting, and more importantly, this study is the first to quantitatively assess childbirth experiences in China. The present study proved that the CEQ-C could be used in facilities to avoid a poor response rate in community settings. We applied some strategies to reduce the possibility that the responses of people who completed these questionnaires in a facility were conditioned by this circumstance to the best of our abilities. First, while conducting the study, trained nurses obtained full informed consent from the participants, and all participants provided oral consent to complete the study. Second, we used online questionnaires to allow the women to complete and submit the questionnaires on their smartphones in a private setting. Third, we informed the women that the staff at the facilities were blinded to the data. Finally, we allowed the women to complete the questionnaire immediately before discharge to prevent their participation from affecting the health care they received. A second strength of this study is its large sample size of 1,747 women from 50 facilities across 4 regions. Third, the forward and backward translation process involved the creator of the CEQ, which helped achieve equivalence with the original version.

The weaknesses of this study are described below. First, some clinical data, such as the labor duration (less or more than 12 h), augmentation (use or no use), and pain relief method (no pain relief, pharmacological or nonpharmacological pain relief), were robustly reported by the women and were not collected from the patient records; thus, these data might be inaccurate, which would have further affected the outcome. More accurate records regarding the precise timing of the onset of labor, the use of intrapartum oxytocin infusion, and the pain relief method would be desirable. Second, an unresponsive bias may exist in our study sample. In our study, the proportion of instrumental deliveries in the population included in this analysis was 7%, a lower value than in other counties but a higher value than in other studies conducted in China.[38] The unresponsive bias means that the exposure of instrumental deliveries in this study group differs from women who were not included in the study and may be related to the situation that women who have experienced instrumental deliveries pay more attention to themselves and are readily complete the questionnaire when they receive an invitation to participate in the survey. This bias may have impacted the outcome of the women’s childbirth experience score. Third, future studies should include women with cesarean section deliveries and determine the percentage of cesarean sections performed at each facility to improve our understanding of this situation in China.

## Conclusions

In summary, a Chinese version of the CEQ (CEQ-C) was produced and a psychometric validation of this instrument was conducted in the present study. The CEQ-C was a reliable and valid tool that was administered to assess childbirth experiences in mainland China. The present study supports the use of the CEQ-C in health facility settings, providing an efficient and cost-effective way for health care providers to rapidly obtain an overview of the quality of intrapartum care from women’s perspectives, identify the needs of women and improve the quality of maternity care. In addition, the adjusted CEQ-C might facilitate future research investigating childbirth experiences in China and allow comparative studies to be conducted between Western and Eastern settings. However, our sample might be inconsistent with the general population; therefore, caution is required when applying the findings of the present study.

## Supporting information

S1 File Childbirth Experience Questionnaire. (DOCX)
