# Supplementary material for: Adaptation of the Childbirth Experience Questionnaire (CEQ) in China: A multisite cross-sectional study
Source: PLoS One. 2019 Apr 24;14(4):e0215373. doi: 10.1371/journal.pone.0215373 (PMC6481804; doi:10.1371/journal.pone.0215373)
Supplement: S1 File — (DOCX) [file pone.0215373.s001.docx]

**S1 file：产妇分娩体验问卷**

**Childbirth experience questionnaire**

分娩护理的目标之一是保证产妇拥有良好的分娩体验。为了了解您本次的分娩体验，特向您发放此问卷。您的回答将为分娩护理的质量评价提供宝贵的信息，希望您能认真回答每个问题。医院的人员不会看到您填写问卷的内容，本次调查不会对您就医造成任何影响，请您如实作答，感谢您的配合！您可以随时终止填写，不会对您造成任何影响。（本调查仅针对自然分娩的产妇，剖宫产产妇可以不用答题，谢谢。）

One of purposes of delivery care is to ensure that women have good experiences. This questionnaire is sent to you for understanding your delivery experience of this time. Your answers will provide precious messages for quality evaluation on delivery care, so we hope that you can answer every question earnestly. Your answer to this questionnaire is blind to staff of the hospital. This survey will not affect your care provided by professional staff in the hospital, so we ask you to answer as per the facts. Thank you for the cooperation! You can give up freely, it doesn’t matter. (This survey only aims at puerperae that have natural deliveries, and those having deliveries by caesarean section may not answer. )

1. 您的分娩方式 [单选题] *

| ○阴道分娩，请您继续回答问题 |
| --- |
| ○剖宫产，您无需回答问题，谢谢！   1. What is your delivery mode? [ Single choice]*   ○ Vaginal delivery. Please continue to answer the next question.  ○ Caesarean delivery. You need not answer. Thank you! |

2. 您现在所在的医院 [填空题] *

_________________________________

1. Which hospital have you been hospitalized now? (Filling the blank)*

______________________________________________

3.您的出生日期 [单选题] *

3.What is your birth date? [ Single choice]*

4. 您的学历： [单选题] *

| ○初中及以下 |
| --- |
| ○高中/中专/技校 |
| ○大专 |
| ○大学本科 |
| ○硕士研究生 |
| ○博士研究生  4.What is your educational degree? [ Single choice]*  ○ Graduated from a junior middle school or below.  ○ Graduated from a senior middle school/ technical secondary school/ technical school.  ○ Graduated from a junior college.  ○ Bachelor.  ○ Master.  ○ Doctor. |

5. 您的工作情况： [单选题] *

| ○国家机关、企事业单位工作人员 |
| --- |
| ○民营企业/外企 |
| ○个体经营 |
| ○务农(农林牧渔) |
| ○在家或待业 |
| ○其他 |

5.What is your occupation? [ Single choice]*

○ Work in a private enterprise/ foreign enterprise.

○ Self-employed.

○ Engaged in agriculture (including agriculture, forestry, animal husbandry and fishery) .

○ Unemployed or waiting for employment.

○ Others.

6.户籍类型： [单选题] *

| ○夫妻双方均为城镇户口 |
| --- |
| ○夫妻双方或一方为农业户籍 |
| ○双方均为农村户籍 |

6. What type is your household registration? *

○ That the couple are both urban household registration.

○ Both or one of the couple are rural household registration.

○ Both of the couple are rural household registration.

7. 您是否参加过医院举办的产前的学习班： [单选题] *

| ○否 |
| --- |
| ○是,参加次数___________________________ . |

7. Whether have you participated in training classes before delivery held by a hospital? [ Single choice]*

○ No.

○ Yes. Participation times: _

**下面一些问题是关于您在这次待产和分娩过程中(从进入待产室待产一直到孩子出生后转入病房前这一段时间)您的一些感受和体会，请您回忆当时的情景，并根据您的真实感受耐心回答，谢谢。**

**The following questions are about your some feelings and experiences during labor and delivery (** **from entering the labor room to before transferring to the postnatal ward after the birth of baby). Please recall then situations and answer in patience. Thank you.**

8. 待产和分娩整个过程和我期望的一样 [单选题] *

| ○非常不同意 | ○大部分不同意 | ○大部分同意 | ○非常同意 |
| --- | --- | --- | --- |

8.Labour and birth went as I had expected.

| ○Totally disagree | ○Mostly disagree | ○Mostly agree | ○Totally agree |
| --- | --- | --- | --- |

9. 待产和分娩过程中我感觉自己有力量 [单选题] *

| ○非常不同意 | ○大部分不同意 | ○大部分同意 | ○非常同意 |
| --- | --- | --- | --- |

9. I felt strong during labour and birth.

| ○Totally disagree | ○Mostly disagree | ○Mostly agree | ○Totally agree |
| --- | --- | --- | --- |

10. 待产和分娩过程中我感觉到害怕 [单选题] *

| ○非常不同意 | ○大部分不同意 | ○大部分同意 | ○非常同意 |
| --- | --- | --- | --- |

10 I felt scared during labour and birth.

| ○Totally disagree | ○Mostly disagree | ○Mostly agree | ○Totally agree |
| --- | --- | --- | --- |

11. 待产和分娩过程中我感觉自己有能力 [单选题] *

| ○非常不同意 | ○大部分不同意 | ○大部分同意 | ○非常同意 |
| --- | --- | --- | --- |

11 I felt capable during labour and birth.

| ○Totally disagree | ○Mostly disagree | ○Mostly agree | ○Totally agree |
| --- | --- | --- | --- |

12. 在待产和分娩过程中我感觉到累 [单选题] *

| ○非常不同意 | ○大部分不同意 | ○大部分同意 | ○非常同意 |
| --- | --- | --- | --- |

12 I was tired during labour and birth.

| ○Totally disagree | ○Mostly disagree | ○Mostly agree | ○Totally agree |
| --- | --- | --- | --- |

13. 在待产和分娩过程中我感觉很幸福 [单选题] *

| ○非常不同意 | ○大部分不同意 | ○大部分同意 | ○非常同意 |
| --- | --- | --- | --- |

13 I felt happy during labour and birth.

| ○Totally disagree | ○Mostly disagree | ○Mostly agree | ○Totally agree |
| --- | --- | --- | --- |

14. 在整个过程中我有很多美好的记忆 [单选题] *

| ○非常不同意 | ○大部分不同意 | ○大部分同意 | ○非常同意 |
| --- | --- | --- | --- |

14 I have many positive memories from childbirth.

| ○Totally disagree | ○Mostly disagree | ○Mostly agree | ○Totally agree |
| --- | --- | --- | --- |

15. 在整个过程中我有很多不愉快的记忆 [单选题] *

| ○非常不同意 | ○大部分不同意 | ○大部分同意 | ○非常同意 |
| --- | --- | --- | --- |

15 I have many negative memories from childbirth

| ○Totally disagree | ○Mostly disagree | ○Mostly agree | ○Totally agree |
| --- | --- | --- | --- |

16. 在待产和分娩过程中，有些事让我觉得很不舒服 [单选题] *

| ○非常不同意 | ○大部分不同意 | ○大部分同意 | ○非常同意 |
| --- | --- | --- | --- |

16 Some of my memories from childbirth make me feel depressed.

| ○Totally disagree | ○Mostly disagree | ○Mostly agree | ○Totally agree |
| --- | --- | --- | --- |

17. 我觉得我待产时(生孩子之前)能决定自己是否能自由活动，包括起床、走动或躺下 [单选题] *

| ○非常不同意 | ○大部分不同意 | ○大部分同意 | ○非常同意 |
| --- | --- | --- | --- |

17 I felt I could have a say whether I could be up and about or lie down.

| ○Totally disagree | ○Mostly disagree | ○Mostly agree | ○Totally agree |
| --- | --- | --- | --- |

18. 我觉得我生孩子的时候能自由选择分娩姿势，包括躺着生、蹲着生或趴在床上生 [单选题] *

| ○非常不同意 | ○大部分不同意 | ○大部分同意 | ○非常同意 |
| --- | --- | --- | --- |

18 I felt I could have a say in deciding my birthing position, including lying, squatting or lying in bed.

| ○Totally disagree | ○Mostly disagree | ○Mostly agree | ○Totally agree |
| --- | --- | --- | --- |

19. 我感觉我选择用哪种方法来缓解疼痛方面有发言权 [单选题] *

| ○非常不同意 | ○大部分不同意 | ○大部分同意 | ○非常同意 |
| --- | --- | --- | --- |

19 I felt I could have a say in the choice of pain relief.

| ○Totally disagree | ○Mostly disagree | ○Mostly agree | ○Totally agree |
| --- | --- | --- | --- |

20. 助产士(或医生/护士)给我提供了充足的陪伴时间 [单选题] *

| ○非常不同意 | ○大部分不同意 | ○大部分同意 | ○非常同意 |
| --- | --- | --- | --- |

20 My midwife devoted enough time to me.

| ○Totally disagree | ○Mostly disagree | ○Mostly agree | ○Totally agree |
| --- | --- | --- | --- |

21. 待产期间陪我的人得到助产士(或医生/护士)足够时间的陪同 [单选题] *

| ○非常不同意 | ○大部分不同意 | ○大部分同意 | ○非常同意 |
| --- | --- | --- | --- |

21 My midwife devoted enough time to my partner.

| ○Totally disagree | ○Mostly disagree | ○Mostly agree | ○Totally agree |
| --- | --- | --- | --- |

22. 在整个过程中，助产士(或医生/护士)告知了我很多的信息，让我知道自己产程进展情况 [单选题] *

| ○非常不同意 | ○大部分不同意 | ○大部分同意 | ○非常同意 |
| --- | --- | --- | --- |

22 My midwife kept me informed about what was happening during labour and birth.

| ○Totally disagree | ○Mostly disagree | ○Mostly agree | ○Totally agree |
| --- | --- | --- | --- |

23. 助产士(或医生/护士)理解我的需要 [单选题] *

| ○非常不同意 | ○大部分不同意 | ○大部分同意 | ○非常同意 |
| --- | --- | --- | --- |

23 My midwife understood my needs.

| ○Totally disagree | ○Mostly disagree | ○Mostly agree | ○Totally agree |
| --- | --- | --- | --- |

24. 我觉的自己在待产和分娩期间得到了助产士(或医生/护士)很好地照顾 [单选题] *

| ○非常不同意 | ○大部分不同意 | ○大部分同意 | ○非常同意 |
| --- | --- | --- | --- |

24 I felt very well cared for by my midwife.

| ○Totally disagree | ○Mostly disagree | ○Mostly agree | ○Totally agree |
| --- | --- | --- | --- |

25. 在待产和分娩期间，这个医院的医护人员技术水平让我觉得很安全 [单选题] *

| ○非常不同意 | ○大部分不同意 | ○大部分同意 | ○非常同意 |
| --- | --- | --- | --- |

25 My impression of the team’s medical skills made me feel secure.

| ○Totally disagree | ○Mostly disagree | ○Mostly agree | ○Totally agree |
| --- | --- | --- | --- |

26. 我觉得我自己很好地掌控了整个待产和分娩过程 [单选题] *

| ○非常不同意 | ○大部分不同意 | ○大部分同意 | ○非常同意 |
| --- | --- | --- | --- |

26 I felt that I handled the situation well.

| ○Totally disagree | ○Mostly disagree | ○Mostly agree | ○Totally agree |
| --- | --- | --- | --- |

27. 总的来说，您觉得的自己生孩子的时候感觉到的疼痛感有多强【0代表一点也没有，100代表非常强烈】*

27 As a whole, how painful did you feel childbirth was?

28. 总的来说，您觉得的自己生孩子的时候感觉到的掌控感有多强【0代表一点也没有，100代表非常强烈】*

28 As a whole, how much control did you feel you had during childbirth?

29总的来说，您觉得的自己生孩子的时候感觉到的掌控感有多强【0代表一点也没有，100代表非常强烈】

29As a whole, how secure did you feel during childbirth?

30. 本次是您第几次分娩 [单选题] *

| ○1 |
| --- |
| ○2 |
| ○3次及以上  30. How many times of deliveries do you have? [ Single choice]*  ○ 1 time.  ○ 2 times.  ○ 3 or more times. |

31. 本次分娩时孕周： [填空题] [填空题]

_________________________________

31. How many gestational weeks did you experienced until the delivery? [ Filling the blank]

______________________________ .

32. 待产期间你住在 [单选题] *

| ○产待一体单间(待产和分娩在一个房间内) |
| --- |
| ○待产单间 |
| ○双人间 |
| ○多人间  32. What room did you stay in during waiting for delivery? [ Single choice]*   - Integrated room for waiting for delivery and delivery. - Single room for waiting for delivery. - Twin room. - Dormitory. |

33. 在待产期间陪同您的人是： [多选题] *

| □无人陪同 |
| --- |
| □丈夫 |
| □母亲或婆婆 |
| □导乐师 |
| □其他 _________________  33. Who accompanied you during waiting for delivery? [ Multiple choices]*   - Nobody. - Husband. - Mother or mother-in-law. - Joy-inducer. - Others______ . |

34. 从出现肚子痛(宫缩)到孩子出生，您大概经历了___小时。 [填空题] *

34. How many hours did you go through about from occurrence of abdominal pains (uterine contraction) to birth of the baby? [ Filling the blank]*

35. 您在产房(待产室及分娩室)里住了大约___小时。 [填空题] *

35. How many hours did you stay about in the delivery rooms (labor and delivery room) ? [ Filling the blank]*

36. 分娩期用药： [单选题] *

| ○无 |
| --- |
| ○有 |
| ○不清楚  36. Was any drug used during delivery? [ Single choice]*  ○ No.  ○ Yes.  ○ Unknown. |

37. 你是否用了无痛分娩： [单选题] *

| ○无，没用任何减痛方法 |
| --- |
| ○有，用了药物麻醉 |
| ○有，用了非药物的方法减轻疼痛 |
| ○其他  37. Whether was any method of painless delivery used for you? [ Single choice]*  ○ No. No method to reduce pains was used.  ○ Yes. Drug anesthesia was used.  ○ Yes. Non-drug methods to reduce pains were used.  ○ Others. |

38. 医生是否给您用了引产的方法(没有自行出现宫缩，利用药物诱发宫缩)： [单选题] *

| ○无 |
| --- |
| ○有 |
| ○不清楚  38. Whether did the physician use any method to induce labor (Under the conditions no automatic occurrence of uterine contraction, relevant drugs were used to induce uterine contraction. ) [ Single choice]*  ○ No.  ○ Yes.  ○ Unknown. |

39. 分娩过程中，医务人员是否给您用了催产方法(产程中用了催产素或人工破膜等加强宫缩的方法) [单选题] *

| ○无 |
| --- |
| ○有 |
| ○不清楚  39. During the delivery process, whether did the medical personnel use any method to expedite the child birth (methods of enhancing uterine contraction like using oxytocin or amniotomy etc.) ? [ Single choice]*  ○ No.  ○ Yes.  ○ Unknown. |

40. 催产方式 [多选题] *

| □催产素 |
| --- |
| □人工破膜 |
| □普贝生 |
| □水囊 |
| □其他： _________________ |
| □不清楚  40. What methods of expediting child birth were used? [ Multiple choices]*   - Oxytocin. - Amniotomy. - Propess. - Water sac. - Others: ______ |

41. 在待产过程中是否用了持续胎心监护： [单选题] *

| ○无 |
| --- |
| ○有 |
| ○不清楚  41. Whether was continuous fetal heart monitor used during waiting for delivery? [ Single choice]*  ○ No.  ○ Yes.  ○ Unknown. |

42. 分娩时的情况： [单选题] *

| ○正常 |
| --- |
| ○产钳助产 |
| ○胎吸 |
| ○臀位牵引 |
| ○其他：_________________  42. How was your situation during delivery? [ Single choice]*  ○ Normal.  ○ By forceps midwifery.  ○ By fetus-extracting midwifery.  ○ By buttock-pulling midwifery.  ○ By other measures: ______ |

43. 会阴情况： [单选题] *

| ○Ⅰ度裂伤 |
| --- |
| ○Ⅱ度裂伤 |
| ○Ⅲ度裂伤 |
| ○完整 |
| ○侧切/正中切 |
| ○不清楚  43. How was your perineum during delivery? [ Single choice]*  ○ I grade laceration.  ○ II grade laceration.  ○ III grade laceration.  ○ Complete.  ○ Lateral incision/ median incision.  ○ Unknown. |

44. 您在待产和分娩过程中有无异常 [单选题] *

| ○无 |
| --- |
| ○有  44. Was there any abnormality during your waiting for delivery and delivery process? [ Single choice]*  ○ No.  ○ Yes. |
